# ‘To give or not to give medication, that is the question.’ Healthcare personnel’s perceptions of factors affecting pro re nata medication in sheltered housing for older adults — a focus-group interview study

**DOI:** 10.1186/s12913-020-05439-4

**Published:** 2020-07-08

**Authors:** Marianne Kollerøs Nilsen, Hege Sletvold, Rose Mari Olsen

**Affiliations:** Faculty of Nursing and Health Sciences, Nord University, Namsos, Norway

**Keywords:** Pro re nata medication, Sheltered housing, Primary care, Healthcare personnel, Older adults, Norway

## Abstract

**Background:**

Residents living in sheltered housing depend on help from healthcare personnel (HCP) with medication management, regarding regular long-term and pro re nata (PRN) medication. The HCP assess the need for PRN medication prior to administration to the residents. The purpose of this study was to describe HCP’s perceptions of factors affecting PRN medication management in sheltered housing for older adults.

**Method:**

This was a qualitative study with five focus-group interviews with 22 HCP working in sheltered housing for older adults. The HCP were heterogenous regarding scholarly education and experiences, working in four different municipalities in mid-Norway, representing urban, sub-urban and rural districts. The analysis was inductive, based on qualitative, manifest, content analysis. The main outcome was HCP perceptions of the factors affecting PRN medication management in sheltered housing.

**Results:**

Four main factors affecting the PRN medication management were identified in the data and were related to either: 1) the medication; 2) the resident; 3) the HCP; or 4) the organisation. These categories included 14 subcategories. Overall, the HCP described the management of PRN medication as a complex process, where the above factors all have impact on the residents’ health and safety.

**Conclusion:**

HCP working in sheltered housing describe that PRN medication management is affected by numerous human factors, that consequently may affect patient outcomes and safety. HCP involved in PRN medication management should be aware of factors that affect their decision-making, and safe management requires a professional practice built on medicines competence, practical skills and experience.

## Background

Medications used ‘as needed’ are given as a response to symptoms(s) that occur without the requirement for regular medication. In long-term care institutions, these medications are given based on observations made by healthcare professionals (HCP). «As needed» (as required) medication is also referred to as pro re nata (PRN) [1–3]. A cross-sectional study among residents living in residential age care services in Australia, found that the median number of PRN on a medication list was 4 (2–6 interquartile range) [4, 5]. In nursing homes, the same number varied, on average between 2 and 4 [1, 6, 7], and the PRN medication in use was significantly varied [1, 7]. In German nursing homes, it was reported that 74.9% of the patients were treated with one or more PRN medications, for which analgesics and psycholeptics were the most often used [6]. In residential age care and homecare patient settings, the most frequent PRN medication used were mild analgesics and laxatives [4].

Medication management may be described as a process that involves the consideration of a patient’s health situation and the need for medication, including the different phases from prescribing to dispensing and administration of medication, and the evaluation of medication effect [3, 8]. The PRN medication management is affected by the HCP at work, and the culture of the clinical environment may influence the choices taken [4, 9]. A case-study from England found that the PRN procedures were open to interpretation, an issue which affected whether medications were given [9]. An Australian study from nursing homes suggested that administrating PRN was too ‘easy’; for example giving tranquilisers to patients, with the aim of producing a calm shift or to avoid disturbing other residents, and that PRN medication is more frequently administered on night shifts [10]. The size of nursing home may affect the amount of PRN medication used [1]. A Norwegian study reports on a general overuse of PRN medication in nursing homes, but occasionally also PRN underuse or misuse [7].

Norway is a decentralised country, and the municipalities differ in terms of demography, economy and geography, all of which can affect the service volume [11]. There are various types of services and facilities based on patients’ individual needs, such as homecare and residential care. Norway provides residential care under institutional care (nursing homes) and sheltered housing (assisted housing). Sheltered housing is heterogenous with respect to staff amount and level of care, factors which will vary in different municipalities. All the sheltered housings have HCP employed, some have 24-h staff, others seeks the residents when they call for assistance. The target population is older people (> 67 years). The resident receives help with daily tasks, including their medication if required. They cook on their own if they are able to, otherwise meals can be served for a fee. By the Norwegian legal regulation, people in sheltered housing live in their own independent home, and rents or buys an apartment from the municipality [11–14]. To our knowledge, PRN medication management in sheltered housing has not been studied in depth.

Studies show that the HCP’s role in decision-making is significant in terms of PRN medication [7, 9, 15], and highlights the need for medication competence among the HCP. Medication competence is a multifaceted combination of knowledge, skills, performance, values and attitudes, and a literature analysis identified 11 areas which medication competence must attend to [15]. PRN medication management requires pharmacotherapeutic competence and patient knowledge [16, 17]. Polypharmacy is common in the elderly population, and aging may alter the drug effects and reactions [18, 19]. Hence, HCP must also observe and monitor the patients’ post-administration of PRN medication [8].

Gaps in the literature point to the requirement for more research about PRN medication management by HCP in sheltered housing. The aim of this study was to describe HCP’s perceptions of factors affecting PRN medication management in sheltered housing for older adults.

## Methods

This study used a descriptive and explorative, qualitative design, utilizing focus-group interviews. Qualitative methods can be useful for identifying and characterising the meaning and understanding of a particular phenomenon [20–22]. A focus-group interview is well suited to identify information about a phenomenon because of the dynamics in a group [23]. Focus groups can uncover factors influencing the topic, and can provide insight into complex tasks [24].

### Study setting, participants and sampling

Five focus-group interviews of HCP working in sheltered housing, from four municipalities representing urban, sub-urban and rural districts in mid-Norway were performed until no new themes were emerging [22]. The time period was from April through November 2018. The sheltered housing had staff members available 24 h a day and were mainly for older adults. The sheltered housing group of residents differed and varied between the municipalities. For example, there were variations in the residents’ frailty, cognition, and grade of dementia, and both the number and dementia of residents varied within one ward. The size of the wards varied from 10 to 35 residents, and in addition, the building sizes and types differed. The sheltered housing organised their personnel in different ways with respect to whether they worked in one or several wards, worked additionally outside the sheltered housing and worked in different shifts.

In sheltered housing, the general medical practitioners (GPs) prescribe the PRN medication for the residents. The responsibility to administer the medication may be delegated to other HCP appointed by the head of unit, who is responsible for ensuring that HCP possess the sufficient competence [3, 8]. The informants were HCP who were certified for medication management (inclusion criteria). HCP included were registered nurses, social educators, health care workers and apprentices in health and social work. Social educators hold a bachelor’s degree and typically work in services for people with learning disabilities and other municipal health and social services. Although their formal education differs from the registered nurses’, they have nursing competencies and skills in medication management [25]. In this study, the definition of ‘nurse’ was used for both registered nurse and social educators because they had the same responsibility of medication administration in the sheltered housing. In principle, mainly nurses manage medications, but other HCP (e.g. health care workers) may also receive the delegated responsibility [3]. To obtain a broad information basis, the focus groups had inter- and intragroup variability, and varied between municipalities. All groups had HCP representing two or more different scholar educations (see Table 1).

**Table 1:** Information about focus groups and informant characteristics

Focus group	Number of informants	Scholarly educations represented	Average number of years employed at this sheltered housing (min-max)	Average years of working experience as HCP (min-max)	Number of residents in the sheltered housing
Interview A ^a^	6	Registered nurse (with advanced education in geriatric) [4] Health care worker [2]	6,8 (5–10)	18,8 (5–29)	15 + 32
Interview B	3	Registered nurse [2] Health care worker [1]	15 (7–22)	13,7 (4–21)	20
Interview C	4	Registered nurse [2] Health care worker [1] Apprentice in health and social work [1]	10,8 (1–28)	11 (1–23)	35
Interview D	5	Registered nurse [1] Social educator [1] Health care worker [3]	8 (2–20)	5,6 (1–10)	25
Interview E	4	Registered nurse [2] Health care worker [1] Apprentice in health and social work [1]	12 (0,3–30)	22,5 (1–32)	15

^a^in focus-group A, two sheltered housing properties were represented, these were located next to each other

For informant recruitment, purposive sampling was chosen [26]. The head of unit recruited HCP to be informants and distributed the information letter and form of consent. In each sheltered housing, 3–6 HCP gave their written consent to study inclusion. To our knowledge, none of the informants that were asked to participate refused the request, and informant dropout during interviews did not occur. The municipality head of department gave their permission to conduct the study. The focus-group interviews were performed at the informants’ workplace.

The first author developed a thematic, semi-structured interview guide (see Additional file 1). The purpose of the interview was to establish an open approach to what influences the PRN medication. A pilot focus-group interview, which is not included in the analysis, was conducted to validate the interview guide. The pilot study informants were nurses with medication management competence, but not working in the municipality.

The moderating team consisted of the first author as moderator, and an assistant moderator writing notes [24]. Only researchers and informants attended the interviews. The preliminary analyses for the first interviews gave new understanding; the questions were modified accordingly for the following interviews, but the frame of the interview guide was the same in all interviews. No repeat interviews were conducted.

A summary of the focus groups and informant characteristics is provided in Table 1. The five interviews were conducted with 22 HCP, with an average work experience of 14.3 years, and 10.5 years in the actual sheltered housing. Each focus-group interview lasted in excess of 1 h (1 h– 1h17m). The interviews were audio recorded and transcribed in verbatim, approved by the informants’ written consent. For organisation, review and analyses, the Nvivo 11/12 computer program was used.

### Data analyses

**Fig. 1:**
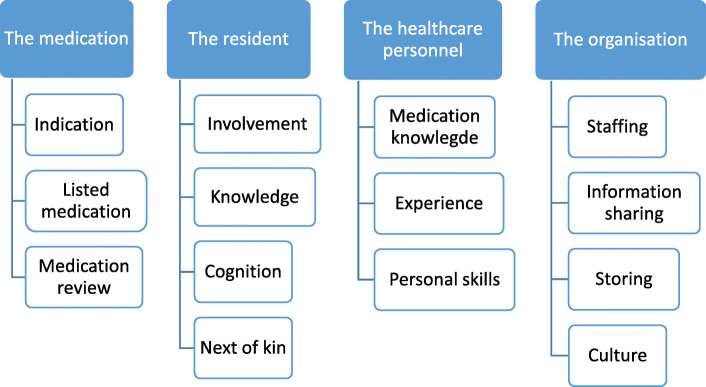
Overview of which factors affect pro re nata medication, perceived by health care personnel working in sheltered housing

The analysis was based on qualitative, manifest, content analysis according to Graneheim and Lundeman [27] and had an inductive approach. Conventional content analysis is the strategy of choice in descriptive qualitative studies [21, 28]. All authors were involved in the analysis process to attend to trustworthiness [28, 29]. The first author transcribed all the interviews verbatim. The first author carried out the analyses from codes to categories. The analysis was performed in several steps. First, the transcripts were divided into content areas related to the main topic raised from the transcripts. Second, the transcripts were divided into meaning units, which comprised sentences or paragraphs related to each other through their content. Then, each meaning unit was condensed and labelled with a code. These codes were abstracted and compared for similarities and differences and sorted in sub-categories, 14 in total. The coding tree is illustrated in Fig. 1 (for a more detailed coding tree see Additional file 2). The subcategories were condensed into four categories. All authors collaborated to create main categories and subcategories in varying levels, with the results being discussed repeatedly. Figure 1 outlines categories with subcategories.

### Trustworthiness

The author group, consisting of two pharmacists and a nurse, considered the study design and analytic approach to be suitable to achieve trustworthiness of the results [27–29]. Participants in this study have various experiences and represent different municipalities to provide a rich variation of information. During regular meetings, the authors ensured and discussed relationship between sampling, data collection and analyses. Categories and sub-categories were regularly discussed, to ensure their relevance.

### Ethical considerations

The study was submitted to the Regional Committees for Medical and Health Research Ethics (REC) in Norway, Ref: 2017/2073/REK midt (Trondheim, Norway), who determined that this research did not require their approval. The Norwegian Centre for Research Data (NSD) was notified, Ref: 57803.

## Results

Through the analysis, four main categories that described the HCP’s considerations about what influences the PRN medication management emerged from the data. These were factors related to: 1) the medication; 2) the resident; 3) the HCP; and 4) the organisation. These categories were elaborated into 14 subcategories (see Fig. 1).

Some of the informants claimed that in their housing it was a broad use of PRN medication, while in others there was only minor use. Those referring to minor PRN usage stated that most of the residents had mild analgesics as regular medication.

The informants understood PRN medication to be every drug given not as regular medication, and referred to analgesics with paracetamol (acetaminophen), some sedatives (i.e zopiclone) and anxiolytics such as Sobril® (oxazepam). Generally, they thought of mild analgesics and anxiolytics as the medication most often used as PRN. The informants stated that they were restrictive with respect to administration of a PRN medication.

### Factors related to the medication

The *indication* for the PRN and the HCPs’ judgement of residents’ symptoms were decisive for PRN administration. The decision-making was more straightforward when the residents’ symptoms were clear or had an obvious cause (e.g. heavy breathing problems or constipation). Administration of hypnotics was made after a critical judgment, and a single night insomnia was not considered to be an indication for PRN hypnotics. Regarding analgesics, the cause of pain was assessed prior to administration, and the decision-making process was relatively difficult in idiopathic pain. Additionally, PRN analgesics were administered prophylactically before nursing.

*‘It’s much easier to give a painkiller when the pain is caused by a fracture or similar, than if it is idiopathic pain where you never find what’s the reason behind.’*

*Listed medications* affected the HCP assessments of medication required by the residents. Medication lists generated by the GP defined which particular medication was allowed to be administered to each resident (general medication lists are not applicable, as used in nursing homes). HCP wanted to follow the medication list, but a conflict in the decision-making process sometimes occurred when the residents’ discontinued drugs were physically available for administration:

*‘Each resident has his or her own medicine basket, and we store each medication until its expiry date. The nametag remains on the medication packaging, although the medicine may be removed from the medication list. [..] it is held on the shelf until the expiry date, and these drugs may be used.’*

Although *medication reviews* are strongly recommended by the Health Authorities they were not a priority in the sheltered housings, which the HCP found to be a matter of concern. Medication reviews could aid in updating of medication lists, and there were examples of PRN medications on the list that should be removed, and medications that were not changed in several decades. The nurses had to take responsibility for updating the medication lists because the GP did not, according to some informants. The number of GPs related to the sheltered housing varied from 2 to 30, and the level of cooperation varied. The HCP monitored changes in medication usage of the residents, and contacted the GP to alter the medication list accordingly:

*‘We had someone using Paracet® (paracetamol/acetaminophen) four times a day for a long time, given after a hip fracture [ … ] we contacted the GP because we found the resident didn’t need it, and suggested it was withdrawn. This was done and it went just fine.’*

Some of the HCP had experience with medication reviews with a pharmacist and found it useful for having an updated medication list (e.g. withdrawing unused medications and changing regular medications to PRN and vice versa). The interprofessional medication review created a consensus of the PRN medication of the resident.

### Factors related to the resident

The residents’ *involvement* was an important issue in the process regarding PRN medication. Most often it was the resident who expressed the PRN need, and the HCP found it important to attend to this request if they found such a need acceptable. However, some of the HCP admitted that they could influence the residents’ wishes, for example by influencing the resident to use paracetamol as an analgesic instead of an opioid. In situations with residents’ using over-the-counter (OTC) medicines which were not on the medication list, the power relation could be the opposite.

‘*We want all responsibility or no responsibility, because it is not justifiable that we sign for the medicine given, since she had so much* (OTC medications) *on her own.*’

According to the informants, some residents were generally concerned about medication use, and did not know their own good. These residents would not necessarily listen to professionals’ justified arguments. The informants indicated that the residents’ autonomy was important, but as HCP they could influence the decision. As one HCP said ‘*We have quite a great impact.’*

A common challenge in the sheltered housing in this study was the increasing number of mentally ill residents. This patient group challenged the HCP’s thoughts about *involvement* because they could become angry if denied their PRN medication. According to the informants, these residents were often demanding (e.g. regarding behavioural problems and decision-making of PRN). Mentally ill residents frequently used PRN medications that the HCP were more restrictive about administering (e.g. benzodiazepines).

The residents’ *knowledge* about their own medication and medication list could affect the PRN administration. The residents knew their PRN possibilities, however knowledge about indications and consequences of frequent use were commonly absent. When HCP provided professionally justified arguments for not giving a particular PRN medication, the resident accepted.

*‘If a medication has effect one day, they (the residents) also want it the next day, without considering the real need for it.’*

The residents’ *cognition* (ability to communicate their own needs) affected the assessment of PRN medication necessity. The informants commented that it was essential to know the residents well in order to develop an awareness of the normal situation and thereby be able to interpret signs for pain or anxiety, for example. Assessment of pain was difficult in cases where residents had trouble expressing their needs and when the cause was not obvious. One informant told they had focus on pain treatment to residents with dementia, they should at least not be in pain. Thus, HCP exercise caution when providing PRN analgesics to demented residents.

*‘If they (the residents) are in pain and need painkillers, and they can’t express themselves, they can behave agitated. It isn’t necessarily psychiatric behaviour, but it looks like that.’*

The *next of kin* and the HCP may have conflicting perceptions and assessments regarding the residents requirement for PRN medication. Often the result would be not to give a PRN medication, argued with HCP knowing the residents’ normal situation better than the next of kin. The residents’ needs were more important than the next of kins’ needs.

*‘We had a resident who got Sobril® (oxazepam) at 10 and 14 o’clock when needed, and if we were not punctual, the spouse became restless, but not the resident.’*

### Factors related to the healthcare personnel

The informants’ *medication knowledge* affected the PRN medication management. Insufficient medication knowledge influenced post-PRN observation and documentation, and informants expressed a desire to quality assure their judgements. The system of delegating medication management responsibility to other personnel than nurses was questioned. An informant said that some nurses used a ‘clinical gaze’ and focused on the reasons behind a particular discomfort, not just relieving it. Pharmacotherapeutic knowledge was important for PRN administration:

*‘Some residents have both lactulose and Imodium® (loperamid) on their medication list and get both simultaneously. The staff’s knowledge can vary’.*

In some situations, the HCP experienced personal internal conflicts when the theoretically correct decision differed from the actual actions performed.

*‘You have to consider the situation [ … ] you would not start a confrontation and sacrifice your own health for a Sobril® (oxazepam). You give it to them even if you know you shouldn’t have.’*

The informants often asked for a second opinion before a final decision was made — this opinion would be complementary to their own competence. *Experience* and practical knowledge were often more important than theoretical knowledge, where experience referred to knowledge about the resident and professional experiences due to many years’ employment in the sheltered housing. Nurses responsible for giving a PRN medication felt an extra obligation to observe and assess by themselves when a newly employed HCP asked for a medication for a resident, but not when it was an experienced college that asked.

*‘It depends who is on duty [..] that they are experienced and know the residents. Then they can predict signals.’ ‘When the nurse is unfamiliar with the residents, there are more narcotics and addictive medication given.’ (Two informants, same focus group).*

The informants had conflicting thoughts regarding how the HCP observed needs and effects of PRN medication. *Personal skills* could contribute to different assessments of observations, however some informants believed assessments were largely the same. Informants did not reflect upon systematics in observations (e.g. the timeframe of pre-PRN observation differed). Priorities among the HCP for using time with the resident, or at least pretend not to be in a hurry, varied.

*‘For some employees it’s easier to give a Sobril® (oxazepam) than try to distract the resident, show them that you have the time and they don’t need it. And maybe you will have a better conscience yourself.’*

### Factors related to the organisation

The system of government, and how the sheltered housing was organised, affected the PRN medication (e.g. there was more use of tranquilisers when staffing was low, according to the informants). The number of HCP and their grade of experience affected the environment in the housing. Regular *staffing* on each shift increased the possibilities of high-quality PRN medication management. In general, the night shifts staffing differed from the daytime staffing, regarding the number and individual HCP present. Some of the informants considered that the threshold for giving medication PRN during night shifts was lower than during the daytime. Another aspect was the holiday seasons.

*‘You feel the uncertainty when the summer begins. You can early recognise the questions: ‘I think it’s something, and some medicine is needed’. It is often persons that are unexperienced in the health care system, and they can be shocked on the reality.’*

Inadequate *information sharing,* both written and oral, affected the PRN medication management. Documentation of PRN medication use patterns, and pre-and-post observations and assessments differed in terms of scope and quality. Quality in documentation was important for taking care of the residents, and the elements within the decision-making process. The administration of a PRN medication was documented, however systematic procedures were absent. Possibilities for oral information sharing between shifts could affect PRN medication management, and informants experienced challenges doing so if the overlap time between shifts was short.

*Storing* of PRN medication affected its accessibility, thereby identified as a contributing factor for PRN management. Residents had medications in their own apartment or locked in a medication- storage at the sheltered housing, with the storing practices varying between municipalities. Where the medications were in storage, the nurses (or someone else delegated with this responsibility) had the key. Then, the HCP had to argue on the resident’s behalf, and had to think through the situation before asking, thereby contributing as a regulating factor for managing PRN.

‘*We have to call someone (a nurse) if we identify a need for PRN medication. Both of us sign to document the reason for taking the medication from storage.’*

Some informants mentioned a change in *culture* regarding PRN medication during recent years, involving a change in focus of the HCP in the sheltered housing to using non-pharmacological interventions prior to giving a medication, an approach which frequently saved them from using PRN medication. Examples of such interventions were conversation as therapy and physical activity, such as hiking.

*‘We have someone (residents) with anxiety, We find that when seeing them, engage in conversation, talking about the wind and weather, or talk a little bit of nonsense, can save us a lot of Sobril® (oxazepam)’.*

In one municipality, they received more resources for an occupational therapist, and the informants found this the best prescription for preventing unrested residents.

*‘We have an occupational therapist and she is really worth the money, we should have several. [ ..] Should really have been on prescription.’*

## Discussion

This study has identified four categories describing factors that affect the PRN medication management in sheltered housing, from the HCP point of view. These were factors related to the medication, resident, HCP and organisation, and represent aspects of professional practice at different levels. Previous studies in similar settings from England, Australia and the US, have outlined comparable findings on decision-making and PRN medication [4, 9, 30]; however, the medication as a contributing factor is novel. Even so, the PRN medications mentioned in this study as the most commonly used (analgesics, hypnotics, anxiolytics and laxatives), are in line with studies from other European countries [6, 9].

The subcategories further describe what affects the PRN medication, and highlights that the medication management and decision-making process concerning PRN is complex. In the practical daily work, this process includes elements from several of the subcategories at the same time (e.g., working as an HCP, the organisation and HCP mutually affect each other [31]). The results demonstrate several aspects that can affect the decision-making process regarding PRN.

The following discussion will highlight the findings we suggest as having substantial implications on the professional practice of HCP in PRN medication management. Nevertheless, the complexity of the PRN medication management process should be kept in mind, and factors not discussed may still have implications of importance in individual patient settings.

The HCP acted in a similar manner as gatekeepers for the residents, and they had significant influence regarding which residents would receive which particular PRN medication. Informants in this study wanted resident involvement, however simultaneously they most often had the decision power of PRN administration. An abuse of this power was not desirable; nevertheless, the decision-making process was influenced by the HCP factors (e.g. the HCP believed that they knew the residents’ best interests). The withholding of prescribed PRN medication can be defined as a medical error [9], but at the same time the HCP have the formal medical competence. This situation exemplifies the paradoxes for HCP in the welfare system [32], and user involvement can be a struggle between autonomy and paternalisation.

The HCP saw themselves as being important spokespersons for the residents, particularly for those who rarely received medical follow-up by their GP. Working with individual human situations as a nurse, knowledge about PRN medication management is not always generalizable, and can be influenced by factors related to both the resident and the HCP. This issue relates to human factors, for example the job, individual and organisational impacts on health and safety-related behaviour as previously described in a WHO report [33]. There are situations where experience is just as important as theoretical knowledge [17, 34]. PRN medication management involves grades of guesswork, supported by medical competence in combination with experience, as described by Lichtner and co-authors [16]*.* The outcome could vary among different HCP. Guidelines can be essential in describing the minimum assessment required in the PRN medication decision-making process, but may be difficult to use [9], or not used, as described in this study. Guidelines could provide less room for action [32], but then again may potentially standardise a service and serve as support in the decision-making process. If guidelines are to be useful, they must be incorporated into the HCP’s daily routines.

The total work experience as HCP, and experience at the specific sheltered housing, varied among the informants in this study. Experience and practical training are important to be able to interpret resident-specific actions, signs and symptoms [15, 30]. Predictability and stability in the staffing situation could affect the residents in sheltered housing and was a contributing factor for HCP when managing PRN. The informants in this study believed that inadequate staffing and with low experience, can lead to more use of PRN medication, and give fewer opportunities for observing the residents and using preventive measures. Working conditions and poor knowledge and skills of staff contributes to medication errors according to an English study from care homes [35]. Workforce amount and education increase the PRN medication administration [4]. Long experience for the actual long-term care home, low staff turnover and familiarity for the housing are important for overall quality of care [36]. Shift and part-time positions and spending little time with the patients make it more difficult to collect relevant information [37]. Policy makers of the health care system should pay attention to HCP staffing, regarding professional competence, medical competence and knowledge, practical skills and experience.

The decision regarding whether to give a particular PRN medication is based on a combination of HCP medication knowledge, experience and skills, a result that is supported by previous studies [9, 15, 16]. These three competence areas could lead to opposite conclusions, and the HCP had to decide which of the competence areas should dominate; sometimes, the medical knowledge had to give way to other reasons. It has been described that nurses require a solid theoretical knowledge base and an ability to transfer the knowledge into professional HCP practice because the situations are often complex [15]. The informants in this study described many competency categories which previously have been defined as being central in the nurses’ medication competence [15]; for example, pharmacology, interdisciplinary collaboration and documentation — the one not mentioned was mathematical and medication calculation.

The increasing number of psychogeriatric illness in long term care [38] may require additional competence, and the head of department should take this in consideration when this patient group is placed in housing where the staff mainly holds geriatric competence.

Collaboration is important for the HCP in the decision-making process of PRN administration, to compensate for uncertainty about their own competence or the wish of not wanting to be held personally responsible. Poor medication competence among nurses, regarding all medications, is described in other studies [39, 40]. To gather a second opinion also makes place for different experiences and emphasises that it is rational to confer with a colleague or collaborative HCP with complementary medical competence and experience. There is a decision-making hierarchy [9], where someone, often a nurse, has the responsibility for what is done. During decision-making, HCP are dependent on detailed information regarding the resident, including the resident’s normal situation. In this study, the collaboration was conducted without focus on the hierarchy, even if it was the nurse who had the responsibility.

Poor information exchange may contribute to medical errors. A qualitative study from Australian residential aged care facilities found that lack of communication channels and incomplete medication lists can be an obstacle to safe medication management and contribute to gaps in the information exchange [41]. Evaluation of effects and adverse reactions is essential for safe medication management within the health service, and the legislation demands documentation [42]. To obtain a real picture for the need of PRN, the HCP depend on each other regarding communication, including documentation. This area should be addressed by the head of department and individual HCP in sheltered housing.

If a PRN medication is given regularly, the medication should be considered being given as a regular regimen and if there is a requirement for a medication review. This study found that HCP called for medication reviews and the GP to take responsibility for updating the residents’ medication lists. In Norway, the patients in nursing homes are supposed to have annual medication reviews [3], and the GPs should perform medication reviews for all older patients who are receiving more than four different medications [43]. It is problematic if no one monitors changes in medication usage and makes changes accordingly (e.g. when PRN become a habit or are used on a regular basis). An inter-professional medication review, like an integrated medication management model [5], which also includes talking with the resident about the medication regime, could be a solution to ensure correct medication prescription and medication lists. Inter-professional medication reviews are challenging to conduct but may improve practice and quality of drug management [37]. A Norwegian study shows that interdisciplinary medication reviews set focus on the importance of good documentation and awareness of symptoms that could be linked to medication use. The results are improved insight and new consciousness when interpreting patients [37]. Medication reviews could be headed by someone outside the sheltered housing (e.g. a pharmacist), who could perform preparatory work and contribute with complementary medication competence. Such intervention may be resource demanding with respect to time and money, and might be difficult for the municipality to prioritise.

Documentation of the reasons for PRN administration including symptoms, and post-administration effects including side effects, are important in order to ensure patient safety [1, 7, 9]. A study from mid-Norway reported that nurses do not consider the consequences of insufficient documentation regarding further medication use and patient safety, and observation is not a main concern in a hectic working environment [37]. The informants in this study had various experiences of PRN documentation, regarding both quantity and quality. Moreover, they were aware of the importance for documenting their work due to follow-up of the PRN medication.

The medication management system in sheltered housing is regulated, and the HCP can only administer medications prescribed on the resident’s medication list. This study describes that HCP PRN administration generally is based on medication lists, although occasionally they act beyond their area of authority and create their own systems. The generation of a system within systems in organisations as a response to a difficult situation is known [31], but multiple and varied, non-integrated systems for medication management can result in medical errors [35]. The assessments the HCP do, depend on which possibilities they have. An Australian study from aged care services emphasises that a high number of medications on the list could be beneficial for the HCP, to have medical treatment options in case of a sudden resident need. Many medications on the list are often seen as an indication of medication review, but it may reflect the GP’s wish to give choices in PRN medication [4]. On the other hand, this issue could make the decision-making process more complex for the HCP. Importantly, medication lists should stay updated with the medicines in current use.

The HCP in this study described institutional power and responsibility, but the sheltered housing are legislated as the resident’s homes, in contrast to a primary health care institution. Thereby, the HCP have a narrow scope of action, but they experience many of the same challenges as an institution (e.g. nursing home). A possibility is to have medications on stock, as is the case in Norwegian nursing homes [2], an approach which may prevent systems within the systems. It could be general lists of medications allowed to store and administrate when required. Having access to PRN medications provides HCP and residents to have flexibility and control, without having to contact the GP. Importantly, such a system would also give the nurses a greater responsibility and competence requirements. It will demand more time for symptom clarification. Pre- and post- administration observation and documentation, and knowledge of residents and medications will be even more important. Also having fewer, dedicated GPs responsible for all the residents in a sheltered housing, could make the PRN medication management process less complex for the HCP, and might be a tool for the correct and safe use of drugs among these patients.

The organisation culture affects the medication management and the resident [9]. In this study, a change in culture was described occurring over the last years, involving an increased focus on using non-pharmacological treatment interventions when possible. Patient safety is a health policy priority in Norway, and the national campaign ‘In Safe Hands’ has been ongoing since 2011 [44] and may explain the medication management culture change. The effective and safe use of non-pharmacological interventions requires sufficient available staff, and such preventive measures are timesaving compared to handling of difficult situations due to behaviour of the residents, according to the informants in this study.

It is not studied in what way these factors actually lead to more or less use in practice, or which of these factors are the most important. This area requires additional research in terms of both qualitative and quantitative studies.

## Strengths and limitations

The aim of this study was to gather varied information about what affects PRN medication management. However, the data were collected from Mid-Norway and may not be representative for all sheltered housing. Nevertheless, we consider the results to be recognisable and transferable to similar housing in different geographical areas. The transferability of this study may be influenced by the various ways to structure the care for older adults in sheltered housing or equivalent in primary health care, both in Norway and internationally. Despite the specific health care context and selection of informants, the study has identified challenges in the health care system that are relevant in similar settings.

The sheltered housing head of unit asked HCP to conduct the study, an issue which may have led to an unspoken pressure to attend the focus groups. Volunteerism was emphasised by the moderator and the written consent scheme, and the study participants had the opportunity to withdraw their consent at any time.

The informants were entirely women, although we acknowledge that including men could have enriched the data material. However, in sheltered housing there are mainly women staffed, and because of the purposive sampling of informants, male HCP informants were not admitted.

The group assembly may affect the focus-group interviews [24]. The researchers defined the inclusion criteria, then had no further impact on selecting the informants. The groups were heterogeneous regarding the informants’ education, percentage of employment and level of responsibility in the sheltered housing. This variability could have affected the dynamics of the group; for example, with respect to possible conflicts of interest, the informants could moderate their statements. Only a few of the informants had focus-group interview experience — some of them were sceptical, and one cannot disregard the so-called ‘researcher reactivity ’ issue [20].

The focus-group moderator was by no means associated with the municipalities or the sheltered housing, a fact which could be both a strength and a limitation of the study. The preunderstanding was not affected by the culture of the municipality or sheltered housing. The moderator background as a pharmacist may have created a distance to the informants, by holding a differing professional focus and sparse internal knowledge of the context and HCP. Respondent validations, including transcript returned to informants, were not performed since we did not collect informant contact information in the consent form, and the communication went through the head of unit.

## Conclusions

This study describes four main areas that affect PRN medication management, according to HCP working in sheltered housing for older adults. These factors are related to the medication, the resident, the health care personnel and the organisation. In general, these factors can be described through human factors because they refer to individual aspects of the HCP and resident, the HCP job in general and the organisation, and they all have impact on the residents’ health and safety. PRN medication management in general, and in sheltered housing in particular, is a complex task, emphasised by the numerous factors found to affect the process. Hence, safe PRN medication management by HCP requires a professional practice with a high degree of medical competence and knowledge, practical skills and experience, in combination with skills in communication and documentation. Furthermore, PRN medication management is affected by HCP working relationships with other staff including GPs, as well as interactions with residents and their relatives, an issue which subsequently may affect residents’ outcomes. An inter-professional health care team including nurses, GPs and pharmacists should be involved in the safe management of PRN medication, for example through medication reviews. An effective system-level approach to support HCP in PRN decision-making, effect assessment, reporting and documentation, is required, and should be incorporated into continuous quality improvement work. Thereby, the HCP professional practice may improve, with the aim of reducing medication errors and adverse drug reactions and contribute to patient safety.

## Supplementary information

**Additional file 1.**

**Additional file 2.**

## Data Availability

The datasets generated and analysed during the current study are not publicly available due to the lack of consent from the informants to share this data, but analysed and aggregated data are available from the corresponding author on reasonable request.

## Abbreviations

PRN: Pro Re Nata
GP: General Practitioner
HCP: Health Care Professionals
OTC: over-the-counter
