# Supplementary material for: ‘To give or not to give medication, that is the question.’ Healthcare personnel’s perceptions of factors affecting pro re nata medication in sheltered housing for older adults — a focus-group interview study
Source: BMC Health Serv Res. 2020 Jul 8;20:622. doi: 10.1186/s12913-020-05439-4 (PMC7346517; doi:10.1186/s12913-020-05439-4)
Supplement: Supplementary file 1 — Additional file 1. [file 12913_2020_5439_MOESM1_ESM.docx]

**SEMISTRUTURED INTERVIEWGUIDE FOCUS GROUP, ENGLISH**

**Introduction**

Presentation of the project, background and aim. What this collected information would be used for.

Make clear the ethical considerations: it is voluntary and it is possible to withdraw anytime.

Information and getting consent to audio recording. Signing of the informed consent form.

*Start audio recording*

**Background information**

Education, years of experience in health care in general and in this sheltered housing, other experience as health care personnel. (Round, everybody speaking)

**Key questions**

- What is the first thing you think about when you hear ‘PRN medication’? (Round, everybody speaking)
- What do you think when you hear this statement «Use of PRN medication depend on who is on duty”?
  - (Follow up) Who is most often taking the initiative to PRN medication?
- Is it some groups of residents, or situations, that are more or less difficult to handle when it comes to PRN medications?
- What do you think when you hear these statements: «It is not a priority to take time for observation of effects in a hectically day” and “It’s no problem if we don’t document on the PRN medication each time”
- What would you say affect the PRN medication most?
  - (Follow up) How does it affect the job you are supposed to do?
- If I would be a holiday substitute with possibilities to handle out PRN medications but not knowing the routines, which advices would you give me?
  - (Follow up) Is there any written information I could read?

**Closure**

Moderator and assistant moderator summarizes key themes from the interview. Feedback from informants.

- Is there any question missing?

Thank you for your time!

*Stop audio-recording*
