# Supplementary material for: ‘To give or not to give medication, that is the question.’ Healthcare personnel’s perceptions of factors affecting pro re nata medication in sheltered housing for older adults — a focus-group interview study
Source: BMC Health Serv Res. 2020 Jul 8;20:622. doi: 10.1186/s12913-020-05439-4 (PMC7346517; doi:10.1186/s12913-020-05439-4)
Supplement: Supplementary file 2 — Additional file 2. [file 12913_2020_5439_MOESM2_ESM.docx]

**Example of the analysis process; coding tree with statements^[[1]](#footnote-1)^**

| Categories | Sub-categories | Codes | Statements used in the article |
| --- | --- | --- | --- |
| Factors related to the medication | Indication | Symptoms and causes | ‘It’s much easier to give a painkiller when the pain is caused by a fracture or similar, than if it is idiopathic pain where you never find what’s the reason behind.’ |
|  |  | Preventive use |  |
|  |  | Necessary use |  |
|  | Listed medication | Which options | Each resident has his or her own medicine basket, and we store each medication until its expiry date. The nametag remains on the medication packaging, although the medicine may be removed from the medication list.[..] it is held on the shelf until the expiry date, and these drugs may be used.’ |
|  |  | Keep it on the list for safety reasons |  |
|  |  | Want to follow prescription |  |
|  | Medication review | Changes, from regular to prn | ‘We had someone using Paracet® (paracetamol/acetaminophen) four times a day for a long time, given after a hip fracture[…] we contacted the GP because we found the resident didn`t need it, and suggested it was withdrawn. This was done and it went just fine.’ |
|  |  | Changes, from prn to regular |  |
|  |  | Who`s responsibility |  |
| Factors related to the resident | Involvement | Autonomy important | ‘We want all responsibility or no responsibility, because it is not justifiable that we sign for the medicine given, since she had so much (OTC medications) on her own.’ |
|  |  | Influencing the residents |  |
|  |  | OTC medications |  |
|  |  | Demanding residents |  |
|  | Knowledge | What residents knows about their medications | ‘If a medication has effect one day, they (the residents) also want it the next day, without considering the real need for it.’ |
|  |  | What residents know about what`s on the list |  |
|  |  | Habits |  |
|  | Cognition | Residents with dementia | ‘If they (the residents) are in pain and need painkillers, and they can`t express themselves, they can behave agitated. It isn’t necessarily psychiatric behaviour, but it looks like that.’ |
|  |  | Mentally ill residents |  |
|  |  | Communication capabilities |  |
|  | Next of kin | Next of kin | ‘We had a resident who got Sobril® (oxazepam) at 10 and 14 o`clock when needed, and if we were not punctual, the spouse became restless, but not the resident.’ |
| Factors related to the healthcare personnel | Medication knowledge | Pharmacological competence | ‘Some residents have both lactulose and Imodium® (loperamide) on their medication list and get both simultaneously. The staff`s knowledge can vary’.  ‘You have to consider the situation […] you would not start a confrontation and sacrifice your own health for a Sobril® (oxazepam). You give it to them even if you know you shouldn’t have.’ |
|  |  | Delegated responsibility to personnel without education |  |
|  |  | Understanding symptoms |  |
|  |  | Practical versus theoretical competence |  |
|  | Experience | Experience versus educational background | ‘It depends who is on duty [..]that they are experienced and know the residents. Then they can predict signals.’ ‘When the nurse is unfamiliar with the residents, there are more narcotics and addictive medication given.’ |
|  |  | Unexperienced versus experienced employees |  |
|  |  | Knowing the residents |  |
|  | Personal skills | Priorities | ‘For some employees it’s easier to give a Sobril® (oxazepam) than try to distract the resident, show them that you have the time and they don’t need it. And maybe you will have a better conscience yourself.’ |
|  |  | Attitudes |  |
| Factors related to the organisation | Staffing | Number of staff | **‘You feel the uncertainty when the summer begins. You can early recognize the questions: ‘I think it's something, and some medicine is needed’. It is often persons that are unexperienced in the health care system, and they can be shocked on the reality.’** |
|  |  | Organisation of staff |  |
|  |  | Regular staff |  |
|  | Information sharing | Orally | (‘We have both written and oral information-sources, its EPR and reports [..] and the residents have PRN sheets in their apartment [..] We have also a book (notebook) which we use for information.’^[[2]](#footnote-2)^) |
|  |  | Written |  |
|  |  | Systematic documentation |  |
|  | Storing | In the apartment | *‘*We have to call someone (a nurse) if we identify a need for PRN medication. Both of us sign to document the reason for taking the medication from storage.’ |
|  |  | In medication storage |  |
|  | Culture | Allowance to use time with the residents | ‘We have someone (residents) with anxiety, We find that when seeing them, engage in conversation, talking about the wind and weather, or talk a little bit of nonsense, can save us a lot of Sobril® (oxazepam)’.  ‘We have an occupational therapist and she is really worth the money, we should have several.[ ..] Should really have been on prescription.’ |
|  |  | Use of non-pharmacological treatment |  |
|  |  | Occupational therapist |  |

1. Because of the amount of data the meaning units and condensations of these are not included [↑](#footnote-ref-1)
2. This statement is not included in the article [↑](#footnote-ref-2)
